# Association of Socioeconomic Status With Risk Factor Target Achievements and Use of Secondary Prevention After Myocardial Infarction

**DOI:** 10.1001/jamanetworkopen.2021.1129

**Published:** 2021-03-10

**Authors:** Joel Ohm, Per H. Skoglund, Henrike Häbel, Johan Sundström, Kristina Hambraeus, Tomas Jernberg, Per Svensson

**Affiliations:** 1 Department of Medicine Solna, Karolinska Institutet, Stockholm, Sweden; 2 Department of Emergency Medicine Solna, Karolinska University Hospital, Stockholm, Sweden; 3 Center for Palliative Care, Stiftelsen Stockholms Sjukhem, Stockholm, Sweden; 4 Unit of Biostatistics, Institute of Environmental Medicine, Karolinska Institutet, Stockholm, Sweden; 5 Department of Medical Sciences, Uppsala University, Uppsala, Sweden; 6 Department of Cardiology, Falu Hospital, Falun, Sweden; 7 Department of Clinical Sciences, Danderyd University Hospital, Karolinska Institutet, Stockholm, Sweden; 8 Department of Clinical Science and Education, Södersjukhuset, Karolinska Institutet, Stockholm, Sweden

## Abstract

**Importance:**

Low socioeconomic status (SES) is associated with poor long-term prognosis after myocardial infarction (MI). Plausible underlying mechanisms have received limited study.

**Objective:**

To assess whether SES is associated with risk factor target achievements or with risk-modifying activities, including cardiac rehabilitation programs, monitoring, and drug therapies, during the first year after MI.

**Design, Setting, and Participants:**

This cohort study included a population-based consecutive sample of 30 191 one-year survivors of first-ever MI who were 18 to 76 years of age, resided in the general community in Sweden, were followed up until their routine 11- to 15-month revisit, and were registered in the national registry Swedish Web-System for Enhancement and Development of Evidence-Based Care in Heart Disease Evaluated According to Recommended Therapies (SWEDEHEART) from 2006 through 2013. Data analyses were performed from January to August 2020.

**Exposure:**

Individual-level SES by proxy disposable income quintile. Secondary exposures were educational level and marital status.

**Main Outcomes and Measures:**

Odds ratios (ORs) with 95% CIs for achieved risk factor targets at the 1-year revisit and for use of guideline-recommended secondary prevention activities.

**Results:**

The study comprised 30 191 participants (72.9% men) with a mean (SD) age of 63.0 (8.6) years. Overall, higher SES was associated with better target achievements and use of most secondary prevention. The highest (vs lowest) income quintile was associated with achieved smoking cessation (OR, 2.05; 95% CI, 1.78-2.35), target blood pressure levels (OR, 1.17; 95% CI, 1.07-1.27), and glycated hemoglobin levels (OR, 1.57; 95% CI, 1.19-2.06). The highest-income quintile was associated not only with participation in physical training programs (OR, 2.28; 95% CI, 2.11-2.46) and patient educational sessions (OR, 2.29; 95% CI, 2.12-2.47) in cardiac rehabilitation but also with more monitoring of lipid profiles (OR, 1.20; 95% CI, 1.08-1.33) and intensification of statin therapy (OR, 1.22; 95% CI, 1.11-1.35) during the first year after MI. One year after MI, the highest-income quintile was associated with persistent use of statins (OR, 1.26; 95% CI, 1.10-1.45), high-intensity statins (OR, 1.10; 95% CI, 1.00-1.21), and renin-angiotensin-aldosterone system inhibitors (OR, 1.27; 95% CI, 1.08-1.49).

**Conclusions and Relevance:**

Findings indicated that, in a publicly financed health care system, higher SES was associated with better achievement of most risk factor targets, programs aimed at lifestyle change, and evidence-based drug therapies after MI. Observed differences in secondary prevention activity may be a factor in higher long-term risk of recurrent disease among individuals with low SES.

## Key Points

**Question:**

Is socioeconomic status associated with 1-year target achievements and preceding secondary prevention activities after myocardial infarction?

**Findings:**

In this nationwide cohort study of 30 191 survivors of first myocardial infarction, indicators of low socioeconomic status were associated with worse risk factor target achievements and with poorer use of secondary prevention. For instance, patients in the highest vs lowest quintile of disposable income had greater odds of smoking cessation and participating in patient educational sessions in cardiac rehabilitation.

**Meaning:**

Observed disparities in target achievements and secondary prevention activities may be associated with worse long-term prognosis after myocardial infarction among individuals with lower socioeconomic status.

## Teaser

This nationwide cohort study conducted in a country with a public health care system assesses whether socioeconomic status is associated with risk factor target achievements or with risk-modifying activities among community-dwelling adults in the year after surviving first-ever myocardial infarction.

## Introduction

Socioeconomic status (SES), especially by proxy disposable income, is associated with recurrent major cardiovascular events after a myocardial infarction (MI).^1^ Suggested underlying mediators include SES inequalities in access to acute and secondary prevention treatments^2^ and patient nonadherence with treatment,^3^ but overall the association of SES with secondary prevention remains to be determined.^4,5^ In general, 1-year target achievements for secondary prevention risk factors need improvement^6,7^ and may be even more discouraging among individuals with low SES owing to poorer secondary prevention activity use.

Therefore, we studied achievements of risk factor targets and use of secondary prevention activities associated with SES during the first year after MI in a nationwide cohort of patients who had survived an MI. We hypothesized that SES is associated with risk factor targets at the 1-year revisit as well as secondary prevention activities during the first year after MI.

## Methods

### Study Design, Sample, and Data Sources

This was a nationwide cohort study of patients who survived first-ever MI and attended a routine 11- to 15-month follow-up revisit between January 1, 2006, and December 31, 2013. Data were analyzed from January to August 2020. Study participants were 18 to 76 years of age and included individuals who presented at 70 of 72 cardiac emergency care hospitals throughout Sweden and were in the national quality registry Swedish Web-System for Enhancement and Development of Evidence-Based Care in Heart Disease Evaluated According to Recommended Therapies (SWEDEHEART), which is described elsewhere.^8^ Participant selection is described in more detail in eFigure 1 in the Supplement. The Swedish health care system is public, and, as such, it is equally accessible for all residents. Individual-level data were linked to the study sample from Statistics Sweden, a government agency that annually collects objective socioeconomic data from all Swedish adults,^9^ and from the National Board of Health and Welfare^10^ for outcomes associated with statin dosages based on claimed prescriptions at any Swedish pharmacy. Merging and pseudonymizing registry data were performed by the National Board of Health and Welfare using the unique personal identification number^11^ assigned to each Swedish resident. This study adhered to the relevant Strengthening the Reporting of Observational Studies in Epidemiology (STROBE) reporting guideline.^12^ The study was approved by the Regional Ethical Review Board in Stockholm. Obtaining informed patient consent from pseudonymized data was not feasible, and Swedish law sanctions registration in national quality health registries without written consent. However, all participants were informed of registration and entitled to opt out at any time. Thus, the requirement for obtaining informed consent was waived by the board. No one received compensation or was offered any incentive for participating in this study.

### Socioeconomic Data

Disposable income (mean disposable income per household consumption unit) that accounts for household size and composition was chosen as the principal proxy of SES. It was acquired in the year preceding that of the index MI to avoid misclassification because of sick leave. Income levels were categorized by quintiles into calendar year–specific fifths (lowest referent) because of proportionally higher SES in the registry’s early years and inflation throughout the study period. Because the median income among women was lower than among men, the quintiles were also stratified by sex.

Socioeconomic status is multidimensional, and therefore 2 additional indicators were considered simultaneously in accordance with expert recommendations.^13^ Level of education was categorized as 9 years or less (referent), 10 to 12 years, and more than 12 years based on the highest educational level attained during the year of the 1-year follow-up after MI. Marital status, chosen as an indicator representing social support, was collected at the 1-year revisit and categorized as married (referent) or not married, which included unmarried, divorced, and widowed.

### Definition of Outcomes

Study outcomes were chosen based on established risk factor targets and recommended risk-modifying activities, including cardiac rehabilitation program participation, treatment-guiding monitoring, and use of evidence-based drug therapies.^3,14^ Dependent variables were collected at different points during initial care and at revisits approximately 2 months (range, 4-14 weeks) and at 1 year (range, 11-15 months) after index MI.

Risk factor target achievements were assessed at the 1-year revisit and included smoking cessation among participants smoking at index MI, target levels for weekly physical activity (moderate exertion lasting ≥30 minutes, ≥5 times weekly), low-density lipoprotein cholesterol (LDL-C) (<100 mg/dL before 2012 and <70 mg/dL after 2012; to convert to millimoles per liter, multiply by 0.0259), blood pressure (<140/90 mm Hg), and serum glycated hemoglobin A_1c_ (HbA_1c_) (<7.0% or <53 mmol/mol; to convert percentage of total hemoglobin to proportion of total hemoglobin, multiply by 0.01) levels in study participants with diabetes.

Secondary prevention activities were dichotomous (yes or no) and included participation in programs within cardiac rehabilitation: patient educational sessions, physical training led by a physiotherapist, dietary advice, smoking cessation among current smokers at index MI, and stress management group session if screening at the 2-month revisit indicated any degree of self-assessed anxiety or depression. Participants were considered to have participated in a program if registered at either or both revisits during the first year after MI. Furthermore, repetitive monitoring of HbA_1c_ levels if diagnosed as having diabetes (at least twice during initial care and at the 2-month and 1-year revisit) and lipid levels (at ≥1 revisit) were assessed, and whether statin therapy was intensified between the 2 follow-up visits was recorded. Lastly, drug therapies were assessed and included discharge from initial care with dual antiplatelet therapy, initiation at discharge as well as persistent use at the 1-year revisit of acetylsalicylic acid, statins, renin-angiotensin-aldosterone system (RAAS) inhibitors, and β-blockers. Indication for RAAS inhibitors was defined as diagnosed hypertension, diabetes, or left ventricular ejection fraction of 40% or lower. Indication for β-blockers was defined as left ventricular ejection fraction of 40% or lower at discharge from hospital after index MI. The definitions and management of variables presented in Table 1 are reported in the eMethods in the Supplement.

**Table 1.** Descriptive Characteristics of Participants by Disposable Income Quintiles^a^

Characteristic	No. (%) of participants					
	Lowest quintile	Second quintile	Third quintile	Fourth quintile	Highest quintile	Missing
No. (%) with data	6044	6038	6039	6038	6032	
Sociodemographic						
Educational level, y						687
≤9	2509 (43.5)	2372 (40.5)	2040 (34.4)	1771 (29.7)	1175 (19.7)	
10-12	2571 (44.5)	2779 (47.5)	2850 (48.0)	2884 (48.3)	2496 (41.8)	
≥12	692 (12.0)	703 (12.0)	1042 (17.6)	1314 (22.0)	2306 (38.6)	
Married	2523 (42.0)	3170 (52.8)	3722 (62.0)	3875 (64.4)	4230 (70.4)	156
Year of annual follow-up						0
2006	370 (6.1)	383 (6.3)	385 (6.4)	375 (6.2)	385 (6.4)	
2007	615 (10.1)	590 (9.8)	589 (9.8)	585 (9.7)	582 (9.7)	
2008	760 (12.6)	799 (13.2)	796 (13.2)	805 (13.3)	794 (13.2)	
2009	770 (12.7)	746 (12.4)	753 (12.5)	757 (12.5)	754 (12.5)	
2010	754 (12.5)	729 (12.1)	737 (12.2)	721 (11.9)	728 (12.1)	
2011	828 (13.7)	843 (14.0)	841 (13.9)	846 (14.0)	873 (14.5)	
2012	922 (15.3)	941 (15.6)	940 (15.6)	950 (15.7)	909 (15.1)	
2013	1025 (17.0)	1007 (16.7)	998 (16.5)	999 (16.6)	1007 (16.7)	
Sex						
Female	1639 (27.1)	1636 (27.1)	1636 (27.1)	1636 (27.1)	1633 (27.1)	0
Male	4405 (72.8)	4402 (72.9)	4403 (72.9)	4402 (72.9)	4399 (72.9)	0
Age, mean (SD), y	62.3 (10.1)	64.5 (9.5)	63.1 (8.5)	62.2 (7.4)	62.9 (6.7)	0
Prior risk factor accumulation						
Smoking						785
Never	1802 (30.7)	1982 (33.7)	2028 (34.5)	2011 (34.1)	2379 (40.6)	
Former	1618 (27.5)	1967 (33.4)	1898 (32.3)	2000 (33.9)	2001 (34.2)	
Current	2459 (41.8)	1937 (32.9)	1950 (33.2)	1895 (32.1)	1479 (25.2)	
BMI						3900
≤18.5 (underweight)	52 (1.0)	36 (0.7)	22 (0.4)	33 (0.6)	23 (0.4)	
18.5-25	1491 (28.8)	1482 (28.4)	1515 (28.8)	1398 (26.5)	1661 (31.1)	
25-30 (overweight)	2253 (43.5)	2415 (46.2)	2461 (46.8)	2572 (48.7)	2614 (49.0)	
>30 (obesity)	1387 (26.8)	1290 (24.7)	1266 (24.1)	1279 (24.2)	1041 (19.5)	
Hypertension	2215 (36.9)	2391 (39.9)	2339 (38.9)	2235 (37.2)	2277 (38.0)	193
Diabetes	1000 (16.6)	889 (14.8)	785 (13.0)	701 (11.6)	621 (10.3)	60
Hyperlipidemia	866 (14.4)	921 (15.4)	920 (15.3)	825 (13.7)	878 (14.6)	121
Metabolic syndrome^b^	1724 (36.0)	1609 (33.0)	1628 (32.6)	1644 (33.0)	1427 (28.6)	5569
eGFR, mL/min/1.73 m^2^						1006
≥90	2615 (44.7)	2203 (37.8)	2473 (42.3)	2632 (44.9)	2428 (41.9)	
60-89	2607 (44.6)	2957 (50.7)	2841 (48.6)	2809 (47.9)	2954 (51.0)	
30-59	565 (9.7)	613 (10.5)	490 (8.4)	378 (6.4)	388 (6.7)	
<30	64 (1.1)	60 (1.0)	39 (0.6)	42 (0.7)	27 (0.5)	
History of CHF	73 (1.2)	85 (1.4)	41 (0.7)	45 (0.8)	44 (0.7)	457
Acute presentation and infarct severity						
Main symptom, chest pain	5466 (90.7)	5418 (90.2)	5505 (91.7)	5536 (92.2)	5551 (92.6)	156
Admission ECG ST deviation, STEMI	2739 (51.6)	2650 (49.9)	2665 (50.3)	2678 (50.7)	2607 (50.0)	3775
Admission ECG rhythm, nonsinus	334 (5.6)	393 (6.5)	300 (5.0)	269 (4.5)	280 (4.7)	169
Angiographic findings						3938
MINOCA	503 (9.8)	495 (9.5)	532 (10.1)	518 (9.7)	524 (9.8)	
1-vessel	2289 (44.6)	2396 (46.1)	2547 (48.4)	2655 (49.9)	2665 (49.9)	
2-vessel	1339 (26.1)	1300 (25.0)	1274 (24.2)	1281 (24.1)	1305 (24.4)	
3-vessel or left main	1002 (19.5)	1004 (19.3)	909 (17.3)	869 (16.3)	846 (15.8)	
Troponin maximum quintiles						1833
1 (lowest)	889 (15.7)	923 (16.2)	916 (16.1)	897 (15.8)	933 (16.8)	
2	1043 (18.4)	1092 (19.2)	1110 (19.5)	1122 (19.7)	1036 (18.6)	
3	1105 (19.5)	1166 (20.5)	1177 (20.7)	1158 (20.3)	1134 (20.4)	
4	1234 (21.8)	1189 (20.9)	1203 (21.1)	1196 (21.0)	1213 (21.8)	
5 (highest)	1396 (24.6)	1315 (23.1)	1287 (22.6)	1321 (23.2)	1253 (22.5)	
LVEF, %						4766
≥50	3192 (62.8)	3250 (64.2)	3377 (67.3)	3356 (66.0)	3605 (69.6)	
40-49	1088 (21.4)	1070 (21.1)	965 (19.2)	1060 (20.8)	1003 (19.4)	
<40	799 (15.7)	742 (14.7)	677 (13.5)	669 (13.2)	572 (11.0)	
Coronary interventions						
Angiography performed	5133 (84.9)	5195 (86.0)	5262 (87.1)	5323 (88.2)	5340 (88.5)	0
STEMI angiography	2345 (85.6)	2293 (86.5)	2318 (87.0)	2357 (88.0)	2312 (88.7)	0
NSTEMI angiography	2277 (88.6)	2395 (90.0)	2397 (91.0)	2398 (92.1)	2415 (92.6)	0
PCI if angiographic pathology	3840 (63.5)	3940 (65.3)	3999 (66.2)	4056 (67.2)	4085 (67.7)	0
STEMI PCI (n = 13 339)	2085 (76.1)	2058 (77.7)	2072 (77.7)	2089 (78.0)	2088 (80.1)	0
NSTEMI PCI (n = 13 077)	1425 (55.4)	1542 (57.9)	1564 (59.4)	1584 (60.8)	1604 (61.5)	0
Reperfusion treatment in STEMI						18
No reperfusion	575 (21.0)	554 (20.9)	502 (18.9)	517 (19.3)	499 (19.2)	
Thrombolysis/acute CABG	186 (6.8)	194 (7.3)	215 (8.1)	193 (7.2)	142 (5.5)	
Primary PCI	1975 (72.2)	1899 (71.7)	1945 (73.1)	1964 (73.4)	1961 (75.4)	
Planned procedure referral at discharge	414 (7.8)	427 (8.0)	376 (7.1)	402 (7.6)	369 (7.1)	25
STEMI planned procedure (n = 13 322)	163 (6.0)	182 (6.9)	181 (6.8)	175 (6.5)	171 (6.6)	17
NSTEMI planned procedure (n = 13 069)	251 (9.8)	245 (9.2)	195 (7.4)	227 (8.7)	198 (7.6)	8

Abbreviations: BMI, body mass index (calculated as weight in kilograms divided by height in meters squared); CABG, coronary artery bypass graft; CHF, congestive heart failure; ECG, electrocardiogram; eGFR, estimated glomerular filtration rate; LVEF, left ventricular ejection fraction; MI, myocardial infarction; MINOCA, myocardial infarction with nonobstructive coronary arteries; NSTEMI, non-STEMI; PCI, percutaneous coronary intervention; STEMI, ST-elevation myocardial infarction.

^a^ Disposable income by household consumption unit was stratified by sex and calendar year.

^b^ Including data collected at the 2-month revisit.

### Statistical Analysis

The yearly disposable income in thousands of Swedish crowns was categorized by quintiles for men and women separately. The baseline characteristics of patients are summarized by income quintile, with the values for men and women combined. Continuous variables are presented as mean (SD) values, and categorical variables are presented as positive counts and percentages. The associations between SES and outcomes were evaluated using multivariable logistic regression models for either the full cohort or relevant subgroups. The models included the income quintiles, sex, and the year of the 1-year revisit after MI (baseline) as categorical variables. The causal directed acyclic graph approach (eFigure 2 in the Supplement) was used to obtain bias-minimized estimates of the association of SES with outcomes. Restricted cubic splines with 4 knots were used to adjust for age at baseline. The categorical variables of level of education and marital status were added in a second modeling approach. Thereby, independent associations between mutually adjusted indicators of SES and the outcomes were estimated. No evidence of collinearity between exposure variables was observed. Registry data coverage was typically high, and no missing values were imputed. The characteristics of participants with complete and participants with incomplete outcome data are reported in eTable 1 in the Supplement. Robust sandwich estimators were used to estimate standard errors, and 95% CIs are reported. All calculations and analyses were conducted using Stata, version 16 (StataCorp).

## Results

Application of exclusion criteria (eFigure 1 in the Supplement) rendered a final sample of 30 191 study participants. The mean (SD) age was 63.0 (8.6) years; 8180 participants (27.1%) were women, and 22 011 (72.9%) were men. Patient characteristics at index MI admission are presented in Table 1 by quintile of income stratified by sex and calendar year. Coindicators of SES were associated with one another. Mean age per income quintile was similar, but the age span was greater for individuals with lower rather than higher income. The frequencies of current smoking and obesity were higher in lower-income quintiles. Both diabetes and the metabolic syndrome were more common in participants with lower than higher income, but there was no clear association with prior hypertension, lipid-lowering treatment, kidney dysfunction, or history of congestive heart failure. Nonsinus rhythm apparent on admission electrocardiogram and atypical presenting symptoms were more likely among individuals with low income. The proportion of ST-elevation myocardial infarction (STEMI) and non-STEMI (NSTEMI) was approximately 50% within each quintile of income, but coronary disease was more extensive as assessed by troponin levels, angiographic findings, and higher frequencies of left ventricular dysfunction observed in lower-income quintiles during initial care. Higher income indicated higher rates of patients who underwent angiography, percutaneous coronary intervention if there were pathologic angiographic findings, and primary percutaneous coronary intervention in case of STEMI. Low-income individuals with NSTEMI were more likely to be referred at hospital discharge from index MI to a planned procedural intervention.

### SES and Risk Factor Targets

**Figure 1.**
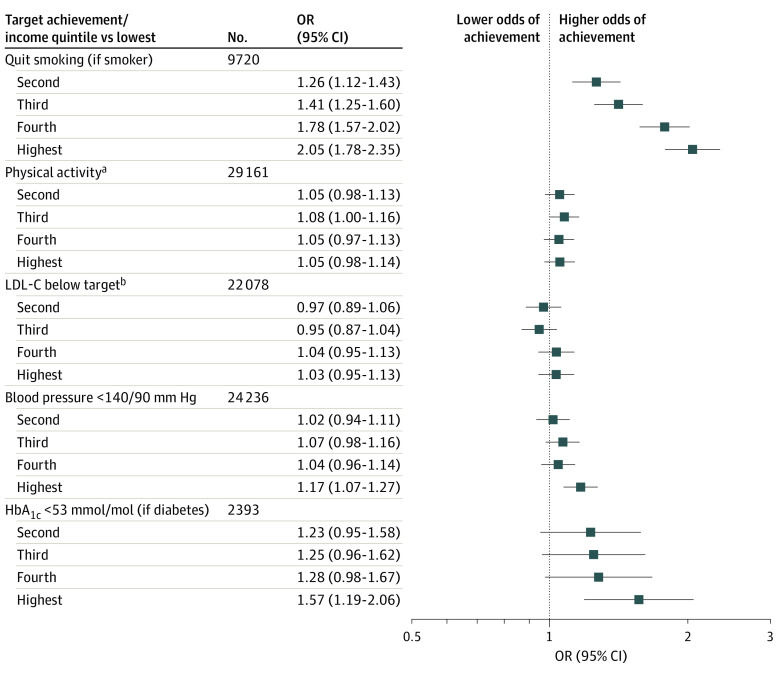
Associations Between Disposable Income Quintiles and 1-Year Risk Factor Target Achievements Associations estimated with odds ratios (ORs) and 95% CIs in logistic regression models adjusted for age, sex, and calendar year. HbA_1c_ indicates hemoglobin A_1c_ (glycated hemoglobin); 53 mmol/mol is equivalent to 7.0% of total hemoglobin (to convert percentage of total hemoglobin to proportion of total hemoglobin, multiply by 0.01). ^a^Moderate or stronger exertion for 30 minutes, 5 times or more per week. ^b^LDL-C indicates low-density lipoprotein cholesterol with a target lower than 100 mg/dL before 2012 or lower than 70 mg/dL after 2012 (to convert to millimoles per liter, multiply by 0.0259).

Target achievement frequencies were typically low in all groups but were worse in lower-income groups. Smoking cessation and target levels for LDL-C and blood pressure were achieved in approximately 50% of patients (Table 2). In multivariable analyses, the highest- vs lowest-income quintile was associated with having quit smoking (odds ratio [OR], 2.05; 95% CI, 1.78-2.35) and blood pressure (OR, 1.17; 95% CI, 1.07-1.27) and HbA_1c_ (OR, 1.57; 95% CI, 1.19-2.06) levels (Figure 1). Associations between mutually adjusted indicators of SES and target achievements are reported in eTable 2 in the Supplement. Only marital status was associated with achieved LDL-C target, and marital status and level of education were associated with targeted weekly physical activity. Associations between disposable income and continuous outcomes, adding routinely measured lipid levels, are reported in eTable 3 in the Supplement.

**Table 2.** Descriptive Data on Risk Factor Target Achievements and Use of Secondary Prevention Activities by Income Quintiles^a^

Achievement or activity	No. (%) of participants					
	Lowest quintile	Second quintile	Third quintile	Fourth quintile	Highest quintile	Missing
Risk factor target achievements						
Quit smoking (smokers at MI [n = 9720])	1197 (48.7)	1060 (54.7)	1115 (57.2)	1187 (62.6)	974 (65.9)	0
Physical activity level (n = 29 161)^b^	2361 (40.8)	2519 (43.1)	2528 (43.2)	2461 (42.1)	2494 (42.7)	1030
LDL-C below target (n = 22 084)^c^	2602 (47.7)	2619 (48.4)	2603 (48.1)	2653 (48.9)	2742 (50.6)	8113
Blood pressure <140/90 mm Hg (n = 24 283)	2967 (49.1)	2931 (48.5)	2931 (48.5)	3023 (50.1)	3189 (52.9)	5955
HbA_1c_ <53 mmol/mol or <7.0% if diabetes (n = 2393)	187 (18.7)	179 (20.1)	166 (21.1)	153 (21.8)	157 (25.3)	1603
Secondary prevention use						
Cardiac rehabiliation program participation						
Physical training program	2009 (33.8)	2252 (37.7)	2727 (45.7)	2913 (48.9)	3214 (54.0)	399
Patient educational session	2262 (38.1)	2701 (45.3)	3145 (52.7)	3325 (55.9)	3518 (59.1)	403
Dietary advice course^d^	923 (15.5)	950 (15.9)	1128 (18.9)	1109 (18.6)	1087 (18.3)	404
Stress management group session	343 (5.8)	350 (5.9)	470 (7.9)	496 (8.3)	585 (9.8)	397
In reported depression or anxiety (n = 10 087)^d^	186 (8.0)	164 (8.1)	225 (11.0)	222 (11.7)	263 (14.8)	1
Smoking cessation program	327 (6.2)	307 (5.8)	319 (6.0)	289 (5.4)	240 (4.6)	3777
In smokers at index MI (n = 9720)^d^	285 (11.9)	248 (13.0)	260 (13.5)	239 (12.9)	184 (12.6)	176
Monitoring						
Screening indicating depression/anxiety	2338 (44.5)	2026 (38.0)	2042 (37.5)	1905 (35.4)	1776 (33.2)	3448
HbA_1c_ measured ≥2/3 opportunities	694 (11.5)	660 (10.9)	672 (11.1)	648 (10.7)	611 (10.1)	0
In diabetes (n = 3996)	324 (32.4)	255 (28.7)	263 (33.5)	233 (33.2)	191 (30.8)	0
Lipid profile measured at any revisit	5086 (84.1)	5085 (84.2)	5135 (85.0)	5117 (84.7)	5218 (86.5)	0
Statin therapy intensification^e^	903 (14.9)	855 (14.2)	983 (16.3)	1003 (16.6)	1113 (18.5)	0
Drug therapies at discharge						
Acetylsalicylic acid	5862 (97.0)	5815 (96.3)	5845 (96.8)	5875 (97.3)	5878 (97.5)	3
Dual antiplatelet therapy	5165 (85.5)	5166 (85.6)	5239 (86.8)	5309 (88.0)	5296 (87.8)	12
Statins	5769 (95.5)	5765 (95.5)	5790 (95.9)	5820 (96.4)	5819 (96.5)	6
High-intensity statin decided at MI discharge^e^	949 (15.7)	965 (16.0)	1021 (16.9)	1026 (17.0)	1005 (16.7)	0
Other lipid-lowering agents	67 (1.1)	77 (1.3)	70 (1.2)	47 (0.8)	70 (1.2)	33
β-Blockers	5624 (93.1)	5545 (91.9)	5584 (92.5)	5574 (92.3)	5522 (91.6)	9
In LVEF <40% (n = 3458)	771 (96.5)	719 (96.9)	647 (95.7)	641 (95.8)	553 (96.7)	1
RAAS inhibitors	4682 (77.6)	4569 (75.7)	4528 (75.1)	4622 (76.6)	4522 (75.1)	37
In LVEF <40%, diabetes, or hypertension (n = 14 816)	2684 (87.5)	2677 (86.8)	2575 (87.0)	2536 (88.2)	2496 (88.1)	24
Drug therapies at 1 y						
Acetylsalicylic acid	5352 (92.6)	5408 (92.6)	5453 (93.1)	5492 (94.0)	5457 (93.5)	1036
Statins	5206 (90.2)	5324 (91.3)	5379 (92.0)	5401 (92.5)	5395 (92.5)	1072
High-intensity statin decided at 1-y revisit^e^	1048 (17.3)	1075 (17.8)	1212 (20.1)	1266 (21.0)	1305 (21.6)	0
β-Blockers if LVEF <40% (n = 3459)	703 (92.9)	662 (92.7)	611 (92.7)	599 (92.2)	517 (92.7)	121
RAAS inhibitors if LVEF <40%, diabetes, or hypertension (n = 14 840)	2546 (86.2)	2604 (87.1)	2493 (86.7)	2449 (87.7)	2447 (89.0)	484

Abbreviations: HbA_1c_, hemoglobin A_1c_ (glycated hemoglobin); LDL-C, low-density lipoprotein cholesterol; LVEF, left ventricular ejection fraction; MI, myocardial infarction; RAAS, renin-angiotensin-aldosterone system.

SI conversion factor: To convert percentage of total hemoglobin to proportion of total hemoglobin, multiply by 0.01.

^a^ Disposable income by household consumption unit was stratified by sex and calendar year.

^b^ Moderate exertion 5 or more times for 30 or more minutes per week.

^c^ LDL-C lower than 100 mg/dL before 2012 and lower than 70 mg/dL after 2012; to convert to millimoles per liter, multiply by 0.0259.

^d^ Rates of participation in part explained by programs not being available at all cardiac care centers.

^e^ Data derived from prescription claims in the national drug registry managed by the Swedish National Board of Health and Welfare.

### SES and Secondary Prevention Activities

Table 2 reports the use of secondary prevention activities from hospital discharge after index MI to the 1-year revisit by income quintile. There were gradients for participation in cardiac rehabilitation programs by higher-income quintiles, ranging from 34% to 54% between the lowest- and highest-income quintiles for participation in physical training programs. This gradient was also observed for stress management group sessions, even though symptoms of anxiety and depression were reported more frequently in lower-income quintiles. Individuals with high income were more closely monitored for lipid levels and were also more likely to have their statin therapy intensified than those with low income. Frequencies of receiving risk-modifying drug therapies at discharge and at 1 year were typically high.

**Figure 2.**
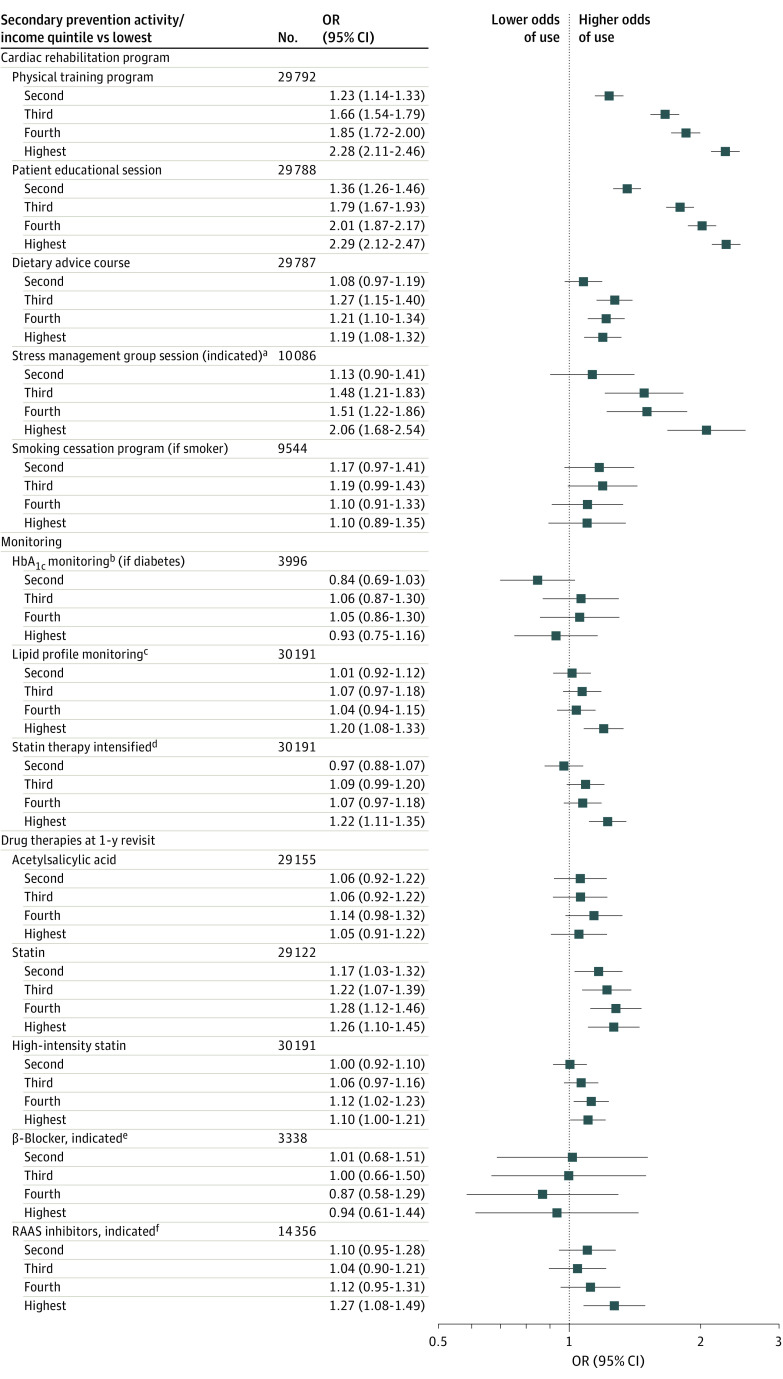
Associations Between Disposable Income Quintiles and Use of Secondary Prevention Activities Associations estimated with odds ratios (ORs) and 95% CIs in logistic regression models adjusted for age, sex, and calendar year. ^a^If reported symptoms of anxiety or depression at the 2-month revisit. ^b^Measured 2 or more times between index myocardial infarction and 1-year revisit; HbA_1c_ indicates hemoglobin A_1c_ (glycated hemoglobin). ^c^At any revisit. ^d^Intensification refers to change of statin therapy intensity to higher category (none, low, moderate, or high) decided at revisits 2 months or 1 year after first myocardial infarction. Data derived from prescription claims in the national drug registry managed by the Swedish National Board of Health and Welfare. ^e^If left ventricular ejection fraction was lower than 40%. ^f^If left ventricular ejection fraction was lower than 40%, or diagnosis of hypertension or diabetes; RAAS indicates renin-angiotensin-aldosterone system.

In multivariable logistic regression models, all indicators of SES were associated with participation in comprehensive cardiac rehabilitation, except in the smoking cessation program. Findings were similar in the subgroup with metabolic syndrome (eTable 2 in the Supplement). The highest-income quintile (vs the lowest) was associated with participation in physical training programs (OR, 2.28; 95% CI, 2.11-2.46), patient educational sessions (OR, 2.29; 95% CI, 2.12-2.47), and stress management group sessions if indicative symptoms (OR, 2.06; 95% CI, 1.68-2.54) and was moderately associated with participation in a dietary advice course (OR, 1.19; 95% CI, 1.08-1.32) (Figure 2). The highest-income quintile, compared with the lowest, was associated with monitoring of lipid profiles (OR, 1.20; 95% CI, 1.08-1.33) as well as intensification of statin therapy (OR, 1.22; 95% CI, 1.11-1.35) between revisits during the first year after MI, whereas no clear associations were observed among participants with diabetes for monitoring of HbA_1c_ level (Figure 2; eTable 2 in the Supplement). Higher income was associated with treatment with dual antiplatelet therapy at hospital discharge (OR, 1.20; 95% CI, 1.08-1.34 in the highest- vs lowest-income quintile). At hospital discharge, only marital status was associated with use of acetylsalicylic acid and with high-intensity statin therapy. Lower level of education and high income were associated with hospital discharge with statin therapy regardless of intensity, whereas only higher level of education was associated with RAAS inhibitor initiation. No associations were observed between indicators of SES and hospital discharge treatment with β-blockers. At the 1-year revisit, all indicators of SES were associated with continued statin therapy (OR, 1.26; 95% CI, 1.10-1.45) and with high-intensity statins (OR, 1.10; 95% CI, 1.00-1.21) for the highest- vs lowest-income quintile. Higher income was further associated with indicated use of RAAS inhibitors (OR, 1.27; 95% CI, 1.08-1.49 in the highest- vs lowest-income quintile) at the 1-year revisit, and being married was associated with the use of acetylsalicylic acid. No associations were observed between SES and the persistent use of β-blockers in the subset of participants with an indication (OR, 0.94; 95% CI, 0.61-1.44).

### Sex Subgroups

The main characteristics and outcomes by income quintiles are reported for men and women separately in eTables 4 and 5 in the Supplement. In general, similar associations between income and the study outcomes were observed. However, the association between the highest-income quintile and achievement of blood pressure target was stronger among women (OR, 1.45; 95% CI, 1.23-1.72) than among men (OR, 1.07; 95% CI, 0.96-1.18). Conversely, men in the highest-income quintile were more likely than those in the lowest-income quintile to be treated with statins (OR, 1.48; 95% CI, 1.24-1.75), whereas no such association was observed among women.

## Discussion

Here we report in a large nationwide cohort study that SES was associated with achieved risk factor targets and the use of a broad range of secondary prevention activities. Our findings may be an explanatory factor of higher long-term risk of recurrent disease among individuals with low SES.^1^ A previous literature review on SES associations with access to cardiac rehabilitation and drug therapies has proven inconclusive.^2^ The present study has several strengths that may contribute substantially to the literature.

### Risk Factor Targets

Target achievements at the 1-year follow-up were typically poor in our cohort but were worse in lower SES groups. We observed associations between higher SES and smoking cessation but also for reaching targeted blood pressure—especially for women—and for HbA_1c_ levels. Our principal proxy for SES, disposable income, was not associated with targeted LDL-C levels or with weekly physical activity. Low rates at target were also observed in the cross-sectional EUROASPIRE IV (European Action on Secondary and Primary Prevention by Intervention to Reduce Events) data collected in the stable phase after a coronary event.^15^ Their convenience sample findings called for urgent strategies to improve guideline adherence. Accordingly, a EUROASPIRE IV–based study suggested intensified secondary prevention for patients with low levels of education.^16^ Our results not only reinforce the need for such strategy but also add estimates for multiple SES indicators, are based on prospectively collected data, and provide a much larger nationwide study sample that includes virtually all patients with an MI at younger than 76 years of age during the study period. Further stressing the urgency for action, the most recent EUROASPIRE V survey reported unchanged or worsened overall target achievements.^17^

### Secondary Prevention Activities

Cardiac rehabilitation and drug therapies are individually associated with the achievement of risk factor targets as well as with improvement of outcomes.^3,14,18^ We report here higher frequencies of smoking and comorbid obesity, diabetes, and metabolic syndrome associated with low SES. Both abdominal obesity^19^ and persistent smoking^20^ are known risk factors for adverse outcomes after MI. We also report here more extensive coronary disease associated with lower SES. All these results are indicative of a greater need for intense secondary prevention. Access to all acute coronary interventions and to most secondary prevention activities was, however, associated with higher SES. This risk-treatment paradox has been described previously but, to our knowledge, never relative to SES.^21^

#### Cardiac Rehabilitation Participation

In this study, SES was associated with participation in a variety of programs in comprehensive cardiac rehabilitation. The participation in physical training programs ranged from 34% to 54% between the lowest- and highest-income quintiles, and associations were also observed for patient educational sessions and stress management group session components. Exercise-based cardiac rehabilitation programs are proven therapies for reducing cardiovascular mortality and reinfarction and for improving health-related quality of life for patients with coronary heart disease.^18,22^ Participation in patient educational sessions alone is also associated with a risk reduction for patients with first-time MI.^23,24^ We report that higher SES was associated with participation in stress management group sessions, although symptoms of depression and anxiety were more prevalent among participants with lower SES. These symptoms have been shown to predict smoking cessation after MI^25^ and are negatively associated with continued adherence to cardiac rehabilitation.^26^ Behavioral therapies lack evidence for improved cardiovascular outcomes but bring other beneficial effects.^27^ Socioeconomic inequalities regarding attendance to cardiac rehabilitation have been previously reported in smaller studies.^28,29^ Possible barriers to participation among individuals with low SES include longer driving distances to health care facilities^30^ and costs related to taking time off work. Providing an extended cardiac rehabilitation program to socially vulnerable patients has been suggested as a strategy to overcome such disparities and improve associated risk factor target achievements.^31^ Increasing cardiac rehabilitation participation has also been shown to be highly cost-effective, with greater potential for health gains and achieved targets among individuals with low SES.^32^

#### Drug Therapies and Monitoring

We observed associations between SES and all risk-modifying drug therapies, except for β-blockers, as well as with statin therapy intensification and treatment-guiding monitoring. Our observed SES disparities regarding drug therapies are relevant to the previously shown paradox of decreasing rates of treatments with higher risk of mortality after MI.^1,33^ A recent study indicated that higher mortality was associated with underuse of antiplatelets, statins, and RAAS inhibitors after coronary artery bypass graft but not with β-blockers.^34^

The association between SES and statin use at 1 year after MI increased with higher income among men but not among women. Greater SES disparities between hospital discharge and the baseline revisit regarding drug therapies were suggestive of poorer adherence for low-income groups. The rate of discontinuation of intended lifelong secondary prevention drug therapy is high in general and increases with time,^34^ and low income was associated with a steeper decrease in the use of dispensed evidence-based drugs during long-term follow-up after coronary artery bypass graft in a recent Swedish study.^35^

Our data are suggestive of more active management from health care professionals because lipid level monitoring improved with higher SES, and statin therapy intensification between revisits was associated with higher income. Stronger advice regarding uptitrating and dosage targets by the time of transfer of care from cardiologists to primary care professionals and integrating dose intensity into secondary prevention performance measures have been identified as possible strategies for improvements.^36,37^ Particularly for women, it may be useful to monitor ambulatory blood pressure to optimize blood pressure control.^38^

### Strengths and Limitations

The main strength of this study was the contemporary, nationwide, and large sample. In addition, access to individual-level data from national registries with high validity^39^ makes results representative for 1-year survivors of first-ever MI in Sweden and in countries with similar populations and health care systems. In countries without universal health coverage, socioeconomic disparities in the medical management may be greater compared with countries with tax-financed health care systems.^2,40^ Therefore, generalization of our findings should be made cautiously and may underestimate health inequities. There is no universal definition of SES, and the relative importance of indicators may vary in different contexts. Alternative indicators of SES, such as rural setting, may carry additional relevant information.^30^ The wealth of data collected allowed us to assess the associations between SES and most risk factor target achievements advised in secondary prevention guidelines.^3,14^ The limitations of all observational studies are the risk of residual confounding and the inability to make causal inferences from observed associations. Important potential registry-related confounding was identified and handled statistically. Cardiac rehabilitation programs for dietary advice, stress management group sessions, and smoking cessation were available for registration for limited periods only and were not available at all follow-up sites. Results for these 3 outcomes should therefore be interpreted with caution because attendance rates may be unrelated to SES and may be less representative.

## Conclusions

Here, we report that low SES, despite public health care in Sweden, is associated with a poorer risk factor target achievement 1 year after first-ever MI and with most target-oriented secondary prevention activities during the preceding year. Unequal use of secondary prevention among individuals with a different SES may be associated with the overall poor target achievements and with increased risk of recurrent disease in low-SES groups. Whether use of secondary prevention mediates the risk of recurrence remains a question for future research.

## Notes

Supplement. **eFigure 1.** Exclusion Flowchart **eFigure 2.** Directed Acyclic Graph **eTable 1.** Descriptive Characteristics in Participants With Complete and Incomplete Data on Secondary Prevention Activity Outcomes **eTable 2.** Association Between Mutually Adjusted Indicators of SES and Risk Factor Target Achievements and Use of Secondary Prevention Activities After Myocardial Infarction **eTable 3.** Association Between Disposable Income Quintiles and Achieved Continuous Risk Factor Levels at 1-Year Revisit **eTable 4.** Sex Specific Descriptive Characteristics at Admission for First Myocardial Infarction by Disposable Income Quintiles **eTable 5.** Sex Specific Associations Between Disposable Income Quintiles and Risk Factor Target Achievements and Use of Secondary Prevention Activities After Myocardial Infarction **eMethods.** Clinical Data Management **eReferences.**
