# Supplementary material for: Association of Socioeconomic Status With Risk Factor Target Achievements and Use of Secondary Prevention After Myocardial Infarction
Source: JAMA Netw Open. 2021 Mar 10;4(3):e211129. doi: 10.1001/jamanetworkopen.2021.1129 (PMC7948055; doi:10.1001/jamanetworkopen.2021.1129)
Supplement: Supplement. — eFigure 1. Exclusion Flowchart eFigure 2. Directed Acyclic Graph eTable 1. Descriptive Characteristics in Participants With Complete and Incomplete Data on Secondary Prevention Activity Outcomes eTable 2. Association Between Mutually Adjusted Indicators of SES and Risk Factor Target Achievements and Use of Secondary Prevention Activities After Myocardial Infarction eTable 3. Association Between Disposable Income Quintiles and Achieved Continuous Risk Factor Levels at 1-Year Revisit eTable 4. Sex Specific Descriptive Characteristics at Admission for First Myocardial Infarction by Disposable Income Quintiles eTable 5. Sex Specific Associations Between Disposable Income Quintiles and Risk Factor Target Achievements and Use of Secondary Prevention Activities After Myocardial Infarction eMethods. Clinical Data Management eReferences. [file jamanetwopen-e211129-s001.pdf]

Supplementary Online Content

Ohm J, Skoglund PH, Häbel H, et al. Association of socioeconomic status with risk factor target achievements and use of secondary prevention after myocardial infarction. *JAMA Netw Open*. 2021;4(3): e211129. doi:10.1001/jamanetworkopen.2021.1129

eFigure 1. Exclusion Flowchart

eFigure 2. Directed Acyclic Graph

eTable 1. Descriptive Characteristics in Participants With Complete and Incomplete Data on Secondary Prevention Activity Outcomes

eTable 2. Association Between Mutually Adjusted Indicators of SES and Risk Factor Target Achievements and Use of Secondary Prevention Activities After Myocardial Infarction

eTable 3. Association Between Disposable Income Quintiles and Achieved Continuous Risk Factor Levels at 1-Year Revisit

eTable 4. Sex Specific Descriptive Characteristics at Admission for First Myocardial Infarction by Disposable Income Quintiles

eTable 5. Sex Specific Associations Between Disposable Income Quintiles and Risk Factor Target Achievements and Use of Secondary Prevention Activities After Myocardial Infarction

eMethods. Clinical Data Management

eReferences.

This supplementary material has been provided by the authors to give readers additional information about their work.

© 2021 Ohm J et al. *JAMA Network Open*.

eFigure 1. Exclusion Flowchart

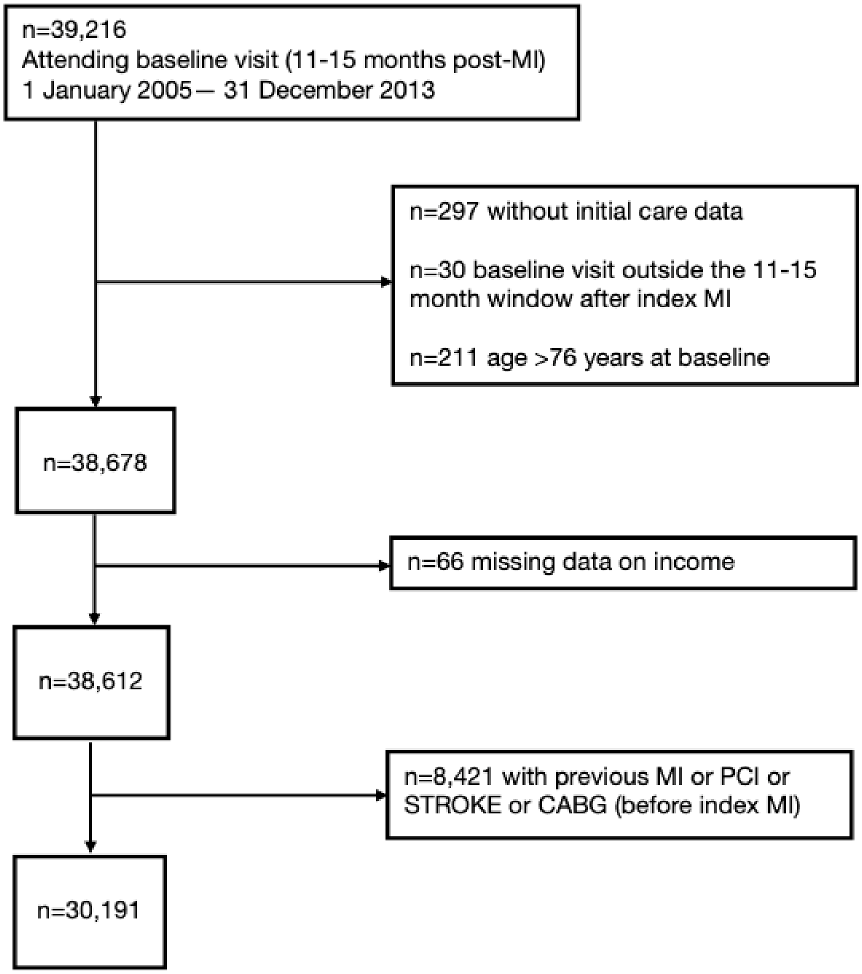

Abbreviations: MI, myocardial infarction; PCI, percutaneous coronary intervention; CABG, coronary artery bypass graft.

© 2021 Ohm J et al. *JAMA Network Open*.

eFigure 2. Directed Acyclic Graph[1]

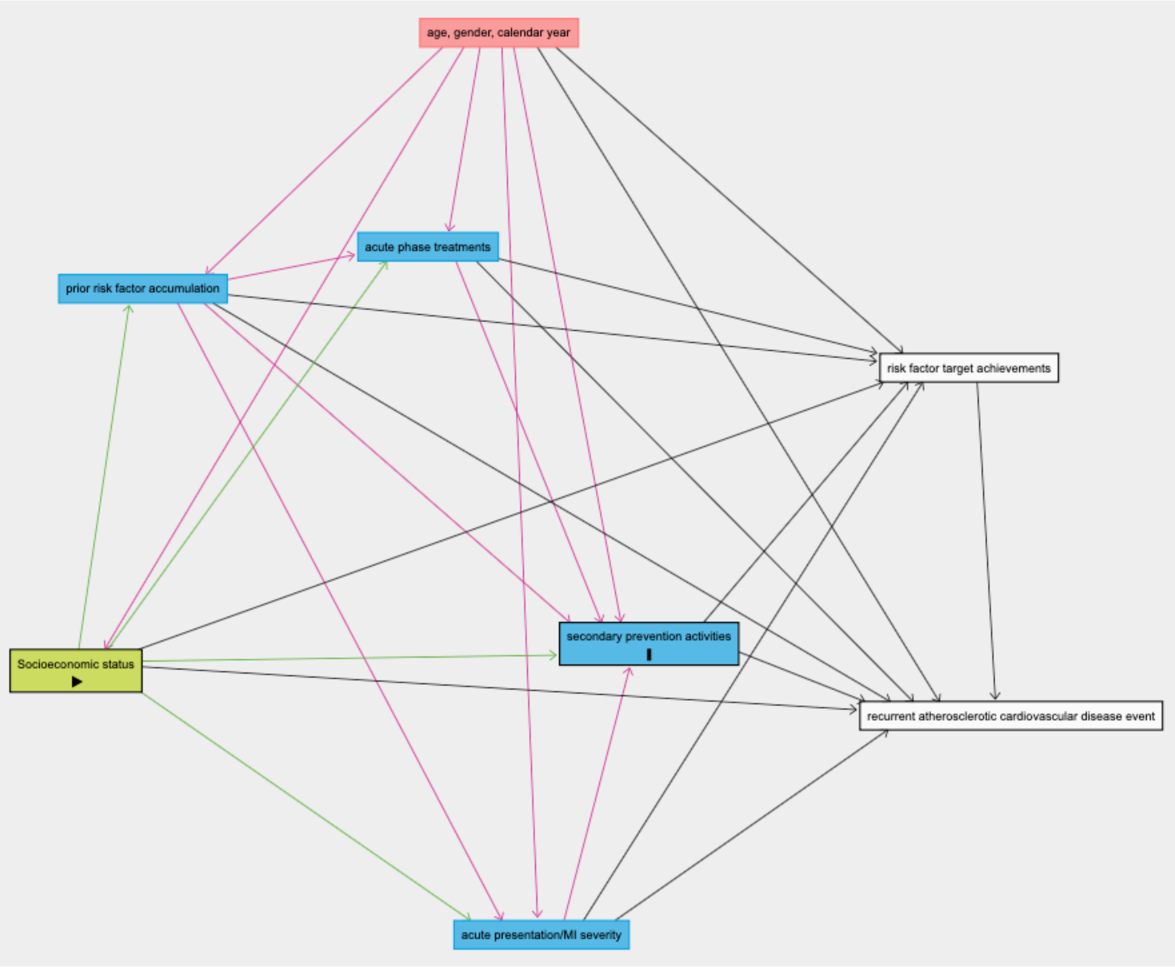

© 2021 Ohm J et al. *JAMA Network Open*.

eTable 1. Descriptive Characteristics of Participants With Complete and Incomplete Data on Secondary Prevention Activity Outcomes

Characteristic	No. (%) of participants		p-value
	≥1 missing outcome	Complete data on outcomes	
No. (%) with data	2,629	27,562	
**Sociodemographics**			
Disposable income[a], quintile			<0.001
Lowest	635 (24.2)	5,409 (19.6)	
Second	585 (22.3)	5,453 (19.8)	
Third	494 (18.8)	5,545 (20.1)	
Fourth	473 (18.0)	5,565 (20.2)	
Highest	442 (16.8)	5,590 (20.3)	
Educational level, y			<0.001
≤9	944 (37.2)	8,923 (33.1)	
10-12	1,105 (43.6)	12,475 (46.3)	
≥12	487 (19.2)	5,570 (20.7)	
Married	1,406 (54.2)	16,114 (58.7)	<0.001
Sex			0.16
Female	743 (28.3)	7,437 (27.0)	
Male	1,886 (71.7)	20,125 (73.0)	
Age, mean (SD), y	63.1 (8.7)	63.0 (8.6)	0.39
Year of annual follow-up, mean (SD), y	2009.5 (2.2)	2010.1 (2.2)	<0.001
**Prior risk factor accumulation**			
Smoking			0.14
Never	907 (35.8)	9,295 (34.6)	
Former	773 (30.5)	8,711 (32.4)	
Current	853 (33.7)	8,867 (33.0)	
BMI, kg/m^2			<0.001
≤18.5 (underweight)	5 (0.2)	161 (0.7)	
18.5-25	498 (23.1)	7,049 (29.2)	
25-30 (overweight)	934 (43.4)	11,381 (47.2)	
>30 (obese)	717 (33.3)	5,546 (23.0)	
Hypertension	1,332 (51.1)	10,125 (37.0)	<0.001
Diabetes[b]	1,624 (61.8)	2,372 (8.6)	<0.001
Hyperlipidemia	708 (27.1)	3,702 (13.5)	<0.001
Metabolic syndrome[c]	730 (45.0)	7,302 (31.7)	<0.001
eGFR, mL/min/1.73m^2			<0.001
≥90	1,124 (44.2)	11,227 (42.1)	
60-89	1,105 (43.5)	13,063 (49.0)	
30-59	267 (10.5)	2,167 (8.1)	
<30	45 (1.8)	287 (0.7)	
History of CHF	44 (1.7)	244 (0.9)	<0.001
**Acute presentation and infarct severity**			
Main symptom, chest pain	2,297 (87.7)	25,179 (91.8)	<0.001
Admission ECG ST deviation, STEMI	1,091 (50.2)	12,248 (50.5)	0.74
Admission ECG rhythm, non-sinus	188 (7.2)	1,388 (5.1)	<0.001
Angiographic findings			<0.001
MINOCA	180 (8.2)	2,392 (9.9)	
1-vessel	929 (42.4)	11,623 (48.3)	
2-vessel	548 (25.0)	5,951 (24.7)	
3-vessel or left main	1532 (24.3)	4,098 (17.0)	
Troponin max, quintile			<0.001
1 (lowest)	478 (19.5)	4,080 (15.8)	
2	503 (20.6)	4,900 (18.9)	
3	510 (20.9)	5,230 (20.2)	
4	508 (20.8)	5,527 (21.4)	
5 (highest)	447 (18.3)	6,125 (23.7)	
LVEF, %			0.06
≥50	1,416 (65.3)	15,364 (66.1)	
40-49	424 (19.5)	4,762 (20.5)	
<40	330 (15.2)	3,129 (13.5)	

Abbreviations: BMI, body mass index; eGFR, estimated glomerular filtration rate; CHF, congestive heart failure; ECG, electrocardiogram; STEMI, ST-elevation myocardial infarction; MINOCA, myocardial infarction with non-obstructive coronary arteries; LVEF, left ventricular ejection fraction.

[a] Disposable income by household consumption unit was stratified by sex and calendar year

[b] More data was needed for participants with diabetes to have complete data

[c] Including data collected at the two-month revisit

© 2021 Ohm J et al. *JAMA Network Open*.

eTable 2. Association Between Mutually Adjusted Indicators of SES and Risk Factor Target Achievements and Use of Secondary Prevention Activities After Myocardial Infarction[a]

	Disposable income quintile[b] vs. lowest				Educational level vs. ≤9 years		Married vs. not
**Achievement or Activity**	**2nd**	**3rd**	**4th**	**highest**	**10 to 12 years**	**>12 years**	**Married**
**RISK FACTOR TARGET ACHIEVEMENTS**							
Quit smoking (if smoker), n=9,553	1.24 (1.10-1.41)	1.30 (1.15-1.47)	1.59 (1.40-1.81)	1.75 (1.52-2.02)	1.02 (0.93-1.11)	1.20 (1.05-1.37)	1.71 (1.57-1.86)
Physical activity level[c], n=28,518	1.04 (0.96-1.12)	1.03 (0.96-1.11)	1.00 (0.92-1.08)	0.98 (0.90-1.06)	1.03 (0.98-1.09)	1.11 (1.04-1.19)	1.12 (1.07-1.18)
LDL-C below target[d], n=21,632	0.97 (0.88-1.06)	0.94 (0.86-1.03)	1.02 (0.93-1.12)	1.02 (0.92-1.12)	1.00 (0.93-1.06)	0.99 (0.91-1.07)	1.08 (1.02-1.14)
Blood pressure <140/90 mmHg, n=23,771	1.01 (0.93-1.10)	1.06 (0.97-1.16)	1.02 (0.94-1.12)	1.12 (1.03-1.23)	1.01 (0.95-1.07)	1.13 (1.04-1.22)	1.04 (0.98-1.10)
HbA1c <53 mmol/mol (If diabetes), n=2,314	1.28 (0.98-1.66)	1.26 (0.97-1.65)	1.26 (0.95-1.66)	1.50 (1.12-2.00)	1.17 (0.96-1.42)	1.27 (0.97-1.66)	1.06 (0.89-1.28)
**SECONDARY PREVENTION USE**							
**Cardiac rehabilitation program participation**							
Physical training program, n=29,121	1.18 (1.09-1.28)	1.52 (1.40-1.64)	1.67 (1.54-1.80)	1.92 (1.77-2.08)	1.20 (1.13-1.26)	1.46 (1.37-1.57)	1.26 (1.20-1.32)
Patient educational session, n=29,117	1.31 (1.21-1.41)	1.67 (1.55-1.80)	1.82 (1.69-1.97)	1.97 (1.82-2.14)	1.31 (1.24-1.38)	1.39 (1.30-1.49)	1.23 (1.17-1.29)
Dietary advice course, n=29,115	1.07 (0.96-1.18)	1.25 (1.13-1.37)	1.18 (1.07-1.31)	1.15 (1.03-1.27)	1.08 (1.00-1.16)	1.07 (0.97-1.16)	1.08 (1.01-1.15)
Stress management group session (if depression/anxiety), n=9,845	1.13 (0.90-1.41)	1.44 (1.17-1.78)	1.39 (1.12-1.73)	1.80 (1.46-2.24)	1.17 (1.00-1.38)	1.63 (1.35-1.96)	1.08 (0.95-1.24)
Smoking cessation program (if smoker), n=9,553	1.17 (0.97-1.41)	1.21 (1.00-1.45)	1.11 (0.92-1.35)	1.13 (0.92-1.40)	1.04 (0.91-1.19)	0.85 (0.69-1.05)	0.94 (0.83-1.07)
Physical training program (if MetS), n=7,886	1.22 (1.06-1.41)	1.39 (1.21-1.61)	1.68 (1.45-1.94)	1.85 (1.58-2.15)	1.21 (1.09-1.34)	1.45 (1.27-1.67)	1.22 (1.11-1.34)
Patient Educational sessions (if MetS), n=7,887	1.43 (1.24-1.64)	1.61 (1.40-1.86)	1.85 (1.60-2.13)	2.16 (1.85-2.52)	1.35 (1.22-1.49)	1.43 (1.24-1.64)	1.15 (1.04-1.26)
Diet course (if MetS), n=7,885	1.06 (0.89-1.26)	1.21 (1.02-1.44)	1.07 (0.90-1.28)	1.20 (0.99-1.45)	1.07 (0.94-1.21)	1.06 (0.89-1.25)	1.07 (0.96-1.20)
**Monitoring**							
HbA1c-monitoring (if diabetes), n=3,862	0.81 (0.66-0.99)	1.03 (0.84-1.27)	1.04 (0.83-1.29)	0.97 (0.77-1.22)	1.07 (0.92-1.25)	0.70 (0.56-0.87)	1.09 (0.94-1.26)
Lipid profile monitoring, n=29,504	1.02 (0.92-1.13)	1.07 (0.96-1.18)	1.03 (0.93-1.15)	1.17 (1.04-1.30)	1.11 (1.03-1.19)	1.14 (1.04-1.26)	0.98 (0.91-1.05)
Statin therapy intensification[e, f], n=29,504	0.95 (0.86-1.06)	1.08 (0.97-1.19)	1.07 (0.96-1.18)	1.23 (1.11-1.37)	1.00 (0.93-1.07)	0.95 (0.87-1.04)	0.98 (0.92-1.04)
**Drug therapies at discharge**							
Acetylsalicylic acid, n=29,501	0.87 (0.71-1.07)	0.91 (0.74-1.13)	1.04 (0.82-1.30)	1.10 (0.86-1.39)	0.93 (0.80-1.08)	0.96 (0.79-1.17)	1.16 (1.01-1.34)
Dual antiplatelet therapy, n=29,492	1.06 (0.95-1.18)	1.11 (1.00-1.24)	1.20 (1.07-1.34)	1.20 (1.07-1.35)	0.96 (0.89-1.04)	0.95 (0.86-1.05)	1.02 (0.95-1.10)
Statins, n=29,498	1.06 (0.88-1.26)	1.10 (0.91-1.32)	1.20 (0.98-1.46)	1.27 (1.03-1.56)	1.03 (0.89-1.18)	0.80 (0.68-0.95)	1.02 (0.90-1.15)
High intensity statin[f], n=29,504	1.02 (0.93-1.12)	1.00 (0.91-1.10)	0.93 (0.84-1.02)	0.96 (0.87-1.07)	1.03 (0.97-1.11)	0.95 (0.87-1.03)	1.07 (1.01-1.14)
Beta blockers (if LVEF <40), n=3,339	1.24 (0.69-2.24)	0.86 (0.49-1.50)	0.79 (0.44-1.41)	1.05 (0.55-1.98)	1.17 (0.77-1.76)	1.14 (0.66-1.94)	0.93 (0.63-1.36)
RAAS inhibitor (if LVEF <40%, HT, or DM), n=14,416	0.91 (0.78-1.07)	0.92 (0.79-1.09)	1.03 (0.87-1.21)	0.97 (0.82-1.16)	1.12 (1.00-1.25)	1.22 (1.05-1.43)	1.00 (0.90-1.11)
**Drug therapies at 1 y**							
Acetylsalicylic acid, n=28,513	1.04 (0.90-1.20)	1.00 (0.86-1.16)	1.09 (0.93-1.27)	0.97 (0.83-1.14)	1.00 (0.90-1.11)	1.03 (0.90-1.18)	1.21 (1.09-1.33)
Statins, n=28,479	1.18 (1.04-1.35)	1.24 (1.09-1.42)	1.35 (1.17-1.55)	1.41 (1.21-1.63)	0.90 (0.81-0.99)	0.70 (0.62-0.79)	1.03 (0.95-1.13)
High-intensity statin[f], n=29,504	0.99 (0.91-1.09)	1.06 (0.96-1.16)	1.11 (1.01-1.23)	1.10 (0.99-1.21)	0.93 (0.87-1.00)	0.92 (0.84-1.00)	1.07 (1.01-1.14)
Beta blockers (if LVEF<40%), n=3,226	1.06 (0.70-1.62)	0.96 (0.64-1.46)	0.85 (0.56-1.27)	0.96 (0.61-1.52)	0.99 (0.73-1.35)	0.88 (0.60-1.30)	0.95 (0.72-1.25)
RAAS inhibitor (if LVEF<40%, HT, or DM), n=13,978	1.08 (0.93-1.26)	1.05 (0.90-1.23)	1.13 (0.96-1.33)	1.27 (1.07-1.51)	0.95 (0.84-1.06)	0.91 (0.78-1.06)	1.02 (0.92-1.13)

© 2021 Ohm J et al. *JAMA Network Open*.

**eTable 2. Association Between Mutually Adjusted Indicators of SES and Risk Factor Target Achievements and Use of Secondary Prevention Activities After Myocardial Infarction (continued)**
Abbreviations: SES, socioeconomic status; LDL-C, low-density lipoprotein cholesterol; HbA_{1c}, hemoglobin A_{1c} (glycated hemoglobin); MetS, metabolic syndrome, LVEF, left ventricular ejection fraction; RAAS, renin-angiotensin-aldosterone system; HT, hypertension; DM, diabetes.<br>[a] Estimated with odds ratios and 95% confidence intervals in multivariable logistic regression models adjusted for age, sex, year of follow-up visit, disposable income, educational level, and marital status. Analyses including all indicators of SES rendered a marginally smaller sample size (n=29,504)<br>[b] Disposable income by household consumption unit was stratified by sex and calendar year.<br>[c] ≥moderate exertion ≥5 times for ≥30 min per week<br>[d] LDL-C <2.5 mmol/L before 2012, LDL-C <1.8 mmol/l after 2012. To convert to mg/dL, multiply values by 38.67.<br>[e] Increased statin therapy intensity (none, low, moderate, high) at two-month or one-year revisit after myocardial infaction.<br>[f] Data derived from prescription claims in the national drug registry managed by the Swedish National Board of Health and Welfare

© 2021 Ohm J et al. *JAMA Network Open*.

eTable 3. Association Between Disposable Income Quintiles and Achieved Continuous Risk Factor Levels at 1-Year Revisit

	Disposable income quintile[a]							
	lowest	second	third	fourth	highest	ANOVA, p	Correlation[b] R	p
Systolic blood pressure, mean (SD), mmHg (n=24,283)	131.5 (17.7)	132.7 (17.6)	131.8 (16.7)	131.7 (16.9)	131.1 (15.9)	<0.001	-0.019	0.004
Diastolic blood pressure, mean (SD), mmHg (n=24,241)	77.0 (10.3)	77.1 (10.0)	77.2 (9.7)	77.6 (9.8)	77.6 (9.4)	0.001	0.017	0.007
LDL-C, mean (SD), mmol/L[c] (n=22,084)	2.30 (0.86)	2.28 (0.83)	2.29 (0.77)	2.27 (0.77)	2.28 (0.74)	0.36	n/a	0.40
HDL-C, mean (SD), mmol/L[c] (n=22,540)	1.21 (0.37)	1.27 (0.37)	1.27 (0.37)	1.29 (0.38)	1.32 (0.38)	<0.001	0.034	<0.001
Total cholesterol, mean (SD), mmol/L[c] (n=22,903)	4.21 (1.03)	4.21 (1.00)	4.23 (0.92)	4.22 (0.94)	4.23 (0.90)	0.87	n/a	0.21
Triglycerides, mean (SD), mmol/L[d] (n=22,477)	1.62 (1.07)	1.52 (0.99)	1.52 (0.95)	1.50 (0.89)	1.41 (0.88)	<0.001	-0.041	<0.001
BMI, mean (SD), kg/m^2, (n=26,305)	27.9 (4.8)	27.6 (4.4)	27.7 (4.4)	27.7 (4.3)	27.1 (4.0)	<0.001	-0.037	<0.001

Abbreviations: ANOVA, analysis of variance; LDL-C, low-density lipoprotein cholesterol; HDL-C, high density lipoprotein cholesterol; BMI, body mass index.
[a] Disposable income by household consumption unit was stratified by sex and calendar year.
[b] R indicates Spearman correlation coefficients, p indicates probability of correlation between coefficients of continuous outcomes and continuous disposable income level.
[c] To convert to mg/dL, multiply values by 38.67
[d] To convert to mg/dL, multiply values by 88.57

© 2021 Ohm J et al. *JAMA Network Open*.

eTable 4. Sex Specific Descriptive Characteristics at Admission for First Myocardial Infarction by Disposable Income Quintiles[a]

	Disposable income quintile									
	lowest		second		third		fourth		highest	
	men	women	men	women	men	women	men	women	men	women
No. (%) with data	4,405	1,639	4,402	1,636	4,403	1,636	4,402	1,636	4,399	1,633
Educational level, y										
≤9	1,792 (42.2)	717 (46.9)	1,715 (39.9)	657 (42.1)	1,526 (35.1)	514 (32.3)	1,340 (30.8)	431 (26.7)	867 (19.9)	308 (19.1)
10-12	1,890 (44.6)	681 (44.5)	1,998 (46.5)	781 (50.1)	2,040 (47.0)	810 (50.9)	2,060 (47.3)	824 (51.0)	1,779 (40.8)	717 (44.4)
≥12	560 (13.2)	132 (8.6)	581 (13.5)	122 (7.8)	776 (17.9)	266 (16.7)	954 (21.9)	360 (22.3)	1,715(39.3)	591 (36.6)
Married	1,999 (45.7)	524 (32.2)	2,517 (57.4)	653 (40.3)	2,818 (64.3)	904 (55.6)	2,883 (65.7)	992 (60.9)	3,062 (69.9)	1,168 (71.8)
Age, mean (SD), y	61.3 (10.0))	65.1 (9.9)	63.6 (9.6)	66.9 (8.6)	62.7 (8.3)	64.4 (8.7)	62.1 (7.3)	62.6 (7.7)	62.9 (6.7)	62.8 (6.8)
Smoking, current	1,848 (43.1)	611 (38.4)	1,339 (31.1)	598 (37.7)	1,318 (30.7)	632 (39.8)	1,282 (29.8)	613 (38.3)	967 (22.6)	512 (32.3)
BMI, kg/m^2										
≤18.5 underweight	18 (0.5)	34 (2.5)	12 (0.3)	24 (1.7)	8 (0.2)	14 (1.0)	6 (0.2)	23 (1.6)	6 (0.2)	17 (1.2)
18.5-25	1,023 (26.9)	468 (33.9)	1,041 (27.1)	441 (31.8)	1,023 (26.5)	492 (35.0)	919 (23.8)	479 (33.8)	1,070 (27.4)	591 (41.2)
25-30 overweight	1,765 (46.4)	488 (35.4)	1,895 (49.4)	520 (37.5)	1,934 (50.1)	527 (37.5)	2,029 (52.5)	543 (38.3)	2,057 (52.7)	557 (38.8)
>30 obese	998 (26.2)	389 (28.2)	888 (23.1)	402 (29.0)	892 (23.1)	374 (26.6)	908 (23.5)	371 (26.2)	772 (19.8)	269 (18.8)
Hypertension	1,473 (33.7)	742 (45.5)	1,621 (37.1)	770 (47.4)	1,590 (36.2)	749 (46.0)	1,548 (35.4)	687 (42.1)	1,618 (37.1)	659 (40.6)
Diabetes	709 (16.2)	291 (17.8)	602 (13.7)	287 (17.6)	536 (12.2)	249 (15.2)	502 (11.4)	199 (12.2)	468 (10.7)	153 (9.4)
Hyperlipidemia	567 (12.9)	299 (18.3)	597 (13.6)	324 (19.9)	606 (13.8)	314 (19.3)	581 (13.2)	244 (15.0)	641 (14.6)	237 (14.6)
Metabolic syndrome[b]	1,232 (35.3)	492 (37.9)	1,115 (31.3)	494 (37.7)	1,122 (30.9)	506 (37.2)	1,173 (32.1)	471 (35.3)	1,019 (28.1)	408 (30.0)
eGFR, mL/min/1.73m^2										
≥90	2,059 (48.3)	556 (35.0)	1,727 (40.6)	476 (30.2)	1,857 (43.5)	616 (39.1)	1,948 (45.7)	684 (42.9)	1,780 (42.0)	648 (41.6)
60-89	1,828 (42.9)	779 (49.1)	2,122 (49.9)	835 (52.9)	2,110 (49.4)	731 (46.4)	2,054 (48.1)	755 (47.4)	2,179 (51.4)	775 (49.7)
30-59	334 (7.8)	231 (14.6)	376 (8.8)	237 (15.0)	278 (6.5)	212 (13.5)	240 (5.6)	138 (8.7)	258 (6.1)	130 (8.3)
<30	43 (1.0)	21 (1.3)	31 (0.7)	31 (1.8)	24 (0.5)	15 (0.9)	25 (0.6)	17 (1.1)	21 (0.5)	6 (0.4)
History of CHF	52 (1.2)	21 (1.3)	62 (1.4)	23 (1.4)	28 (0.6)	13 (0.8)	30 (0.7)	15 (0.9)	25 (0.6)	19 (1.2)

Abbreviations: BMI, body mass index; eGFR, estimated glomerular filtration rate; CHF, congestive heart failure.
[a] Disposable income by household consumption unit was stratified by calendar year.
[b] Including data collected at the two-month revisit

© 2021 Ohm J et al. *JAMA Network Open*.

eTable 5. Sex Specific Associations Between Disposable Income Quintiles and Risk Factor Target Achievements and Use of Secondary Prevention Activities After Myocardial Infarction[a]

	**Disposable income quintile[b] (vs. lowest)**							
	**second**		**third**		**fourth**		**highest**	
	men	women	men	women	men	women	men	women
**RISK FACTOR TARGET ACHIEVEMENTS**								
Quit smoking (if smoker)	1.37 (1.19-1.58)	1.03 (0.82-1.30)	1.46 (1.26-1.69)	1.27 (1.02-1.60)	1.85 (1.60-2.15)	1.58 (1.25-2.00)	2.14 (1.81-2.52)	1.81 (1.41-2.33)
Physical activity level[c]	1.07 (0.99-1.18)	0.96 (0.83-1.10)	1.07 (0.98-1.17)	1.07 (0.93-1.23)	1.01 (0.92-1.10)	1.11 (0.96-1.28)	0.98 (0.89-1.07)	1.19 (1.03-1.38)
LDL-C below target[d]	0.94 (0.85-1.05)	1.04 (0.88-1.24)	0.90 (0.81-1.00)	1.07 (0.91-1.27)	1.04 (0.93-1.15)	1.00 (0.84-1.19)	1.01 (0.91-1.12)	1.05 (0.89-1.25)
Blood pressure <140/90 mmHg	0.95 (0.86-1.05)	1.22 (1.04-1.44)	1.03 (0.93-1.14)	1.17 (0.99-1.37)	1.01 (0.91-1.11)	1.12 (0.95-1.32)	1.07 (0.96-1.18)	1.45 (1.23-1.72)
HbA1c <53 mmol/mol (if DM)	1.26 (0.94-1.70)	1.22 (0.76-1.96)	1.30 (0.96-1.78)	1.17 (0.72-1.88)	1.26 (0.92-1.74)	1.44 (0.86-2.40)	1.68 (1.23-2.30)	1.35 (0.76-2.40)
**SECONDARY PREVENTION USE**								
**Cardiac rehabilitation program participation**								
Physical training program	1.26 (1.15-1.37)	1.15 (1.00-1.33)	1.64 (1.50-1.79)	1.68 (1.45-1.94)	1.84 (1.69-2.01)	1.84 (1.59-2.12)	2.23 (2.04-2.44)	2.35 (2.03-2.73)
Patient educational session	1.39 (1.28-1.52)	1.26 (1.09-1.45)	1.85 (1.70-2.02)	1.63 (1.42-1.88)	2.06 (1.89-2.25)	1.86 (1.61-2.15)	2.28 (2.09-2.49)	2.25 (1.94-2.61)
Dietary advice course	1.09 (0.97-1.22)	1.03 (0.85-1.26)	1.24 (1.11-1.39)	1.35 (1.12-1.62)	1.21 (1.08-1.35)	1.21 (1.00-1.47)	1.15 (1.03-1.30)	1.26 (1.04-1.52)
Stress management if depression/anxiety	1.24 (0.94-1.64)	0.94 (0.65-1.38)	1.52 (1.17-1.98)	1.36 (0.97-1.91)	1.68 (1.29-2.19)	1.20 (0.84-1.72)	1.91 (1.46-2.50)	2.14 (1.53-2.99)
Smoking cessation program (in smokers)	1.20 (0.96-1.50)	1.11 (0.80-1.53)	1.27 (1.02-1.59)	1.06 (0.77-1.46)	1.08 (0.85-1.36)	1.09 (0.79-1.51)	1.15 (0.89-1.47)	1.02 (0.71-1.45)
**Monitoring**								
HbA1c-monitoring (if diabetes)	0.96 (0.76-1.21)	0.64 (0.44-0.92)	1.12 (0.87-1.42)	0.92 (0.64-1.32)	1.07 (0.83-1.37)	0.97 (0.66-1.44)	0.96 (0.75-1.25)	0.80 (0.51-1.23)
Lipid profile monitoring	1.03 (0.92-1.16)	0.96 (0.80-1.16)	1.00 (0.89-1.13)	1.27 (1.04-1.54)	1.00 (0.89-1.12)	1.15 (0.95-1.40)	1.18 (1.04-1.34)	1.25 (1.02-1.53)
Statin therapy intensification[e, f]	0.98 (0.87-1.10)	0.96 (0.80-1.17)	1.11 (0.99-1.25)	1.05 (0.87-1.27)	1.09 (0.97-1.22)	1.07 (0.88-1.30)	1.23 (1.09-1.38)	1.26 (1.04-1.53)
**Drug therapies at discharge**								
Dual antiplatelet therapy	1.06 (0.94-1.20)	1.09 (0.90-1.32)	1.12 (0.99-1.27)	1.14 (0.94-1.38)	1.24 (1.09-1.41)	1.17 (0.96-1.43)	1.20 (1.05-1.36)	1.25 (1.02-1.54)
**Drug therapies at one year**								
Acetylsalicylic acid	1.13 (0.95-1.34)	0.96 (0.75-1.22)	1.09 (0.92-1.30)	1.00 (0.78-1.29)	1.25 (1.04-1.50)	0.94 (0.72-1.22)	1.17 (0.98-1.41)	0.83 (0.64-1.08)
Statins	1.20 (1.03-1.41)	1.11 (0.90-1.37)	1.31 (1.11-1.54)	1.08 (0.87-1.34)	1.37 (1.16-1.63)	1.14 (0.91-1.43)	1.48 (1.24-1.75)	0.99 (0.79-1.24)
High-intensity statin[f]	1.00 (0.90-1.11)	1.00 (0.85-1.18)	1.10 (0.98-1.22)	0.98 (0.83-1.16)	1.14 (1.02-1.27)	1.07 (0.90-1.27)	1.14 (1.02-1.27)	1.00 (0.84-1.19)
Beta blockers if LVEF <40%	1.23 (0.75-2.01)	0.67 (0.32-1.40)	0.89 (0.56-1.41)	1.60 (0.66-3.84)	0.87 (0.54-1.39)	0.89 (0.41-1.93)	0.87 (0.53-1.43)	1.24 (0.49-3.13)
RAAS inhibitors if LVEF <40%, HT, or DM	1.11 (0.92-1.34)	1.09 (0.85-1.41)	1.06 (0.88-1.28)	1.02 (0.79-1.31)	1.15 (0.95-1.40)	1.08 (0.83-1.41)	1.29 (1.05-1.57)	1.27 (0.95-1.68)

Abbreviations: LDL-C, low-density lipoprotein cholesterol; HbA_{1c}, hemoglobin A_{1c} (glycated hemoglobin); DM, diabetes mellitus; RAAS, renin-angiotensin-aldesterone system; LVEF, left ventricular ejection fraction; HT, hypertension.

[a] Estimated with odds ratios and 95% confidence intervals in multivariable logistic regression models adjusted for age, year of follow-up visit.

[b] Disposable income by household consumption unit was stratified by calendar year.

[c] ≥moderate exertion ≥5 times for ≥30 min per week

[d] LDL-C <2.5 mmol/L before 2012, LDL-C <1.8 mmol/l after 2012. To convert to mg/dL, multiply values by 38.67.

[e] Increased statin therapy intensity (none, low, moderate, high) at two-month or one-year revisit after myocardial infaction.

[f] Data derived from prescription claims in the national drug registry managed by the Swedish National Board of Health and Welfare

© 2021 Ohm J et al. *JAMA Network Open*.

eMethods

Clinical data management

Prior risk factor accumulation data was collected at admission for the index MI and are reported in table 1. Body Mass Index (BMI) was calculated by dividing weight (kg) with height (m) squared and categorized according to WHO thresholds. Estimated glomerular filtration rate (eGFR) (ml/min/1.73 m^2) was calculated using the Chronic Kidney Disease Epidemiology Collaboration (CKD-EPI) equation[ref][2] using mean S-Creatinine (µmol/L) and was categorized into CKD staging. The Metabolic syndrome was defined according to the NCEP ATP III 2005 definition[3] with data on collected at the two-month revisit.

Acute presentation and index infarction severity data was collected during initial care. Admission ECG rhythm (sinus, non-sinus) truncated any non-sinus rhythm, Admission ECG ST-deviation (ST-elevation myocardial infarction (STEMI), Non-STEMI) included equivalents to the former such as pathological Q-waves and left bundle branch block configurations. Biomarker max was the highest quintile of the maximum value of measured cardiac biomarkers (Troponin-T, Troponin-I, or high-sensitivity Troponin-T). Angiographic findings data were recategorized (MI with non-obstructive coronary arteries (MINOCA), 1-vessel disease, 2-vessel disease, or 3-vessel or left main obstruction).

Data on coronary interventions were collected during initial care and discharge. Percutaneous coronary intervention (PCI) if angiographic pathology was dichotomized to include any type of PCI. Reperfusion treatment in STEMI was recategorized (none, thrombolysis or acute coronary artery by-pass graft (CABG), or primary PCI). Planned procedure referral at discharge included angiography, PCI, and sub-acute CABG.

© 2021 Ohm J et al. *JAMA Network Open*.

eReferences

1. Textor J, Hardt J, Knuppel S. DAGitty: a graphical tool for analyzing causal diagrams. *Epidemiology.* 2011;22(5):745.
2. Earley A, Miskulin D, Lamb EJ, Levey AS, Uhlig K. Estimating equations for glomerular filtration rate in the era of creatinine standardization: a systematic review. *Ann Intern Med.* 2012;156(11):785-795, W-270, W-271, W-272, W-273, W-274, W-275, W-276, W-277, W-278.
3. Grundy SM, Cleeman JI, Daniels SR, et al. Diagnosis and management of the metabolic syndrome: an American Heart Association/National Heart, Lung, and Blood Institute Scientific Statement. *Circulation.* 2005;112(17):2735-2752.

© 2021 Ohm J et al. *JAMA Network Open*.
